# Dedication to Dr J.M. Zaldívar Comenges (1958–2012)

**DOI:** 10.1016/j.tiv.2017.08.007

**Published:** 2017-12

**Authors:** Maurice Whelan, Andrew Worth, Sandra Coecke, Bas Blaauboer

**Affiliations:** a European Commission, Joint Research Centre, Directorate Health, Consumers and Reference Materials, Chemical Safety and Alternative Methods Unit incorporating EURL ECVAM, Italy; b Institute for Risk Assessment Sciences, Utrecht University, The Netherlands

This special issue entitled “*The Virtual Cell Based Assay*” is dedicated to our former colleague and friend Dr J.M. Zaldívar Comenges, “Josema”, who passed away on 14 September 2012, after a period of prolonged illness. At the time, he was a senior scientist at the European Commission's Joint Research Centre (JRC), where he had been working for over 25 years.

Josema was a brilliant scientist. He had an MSc in Organic Chemistry from the Institut Quimic de Sarria (Universidad Autònoma de Barcelona, Spain), and a PhD in Chemical Engineering from Universiteit Twente (Enschede, The Netherlands). He was a prolific writer and innovator, with more than 300 scientific reports and peer review publications, two patents and five software copyrights to his name.

Josema dedicated his career to the application of advanced mathematical modelling to a wide and diverse range of problems. Examples include computer modelling of chess games, management of traffic flow in Barcelona, safety assessment of chemical reactors, modelling of chemical fate in geochemical, ecological and biological systems, and model-based chemical prioritisation of chemicals based on concern for human health and the environment. During his later years, he channelled his efforts into the development of mathematically-based modelling approaches aimed at improving the safety assessment of chemicals, while also replacing the need for toxicity tests based on animal models. This special issue is a tribute to Josema's pioneering work on the development of a Virtual Cell Based Assay (VCBA), most of which was carried out in the context of the COSMOS project, funded by the EU's 7th Framework Programme.

Josema is remembered by his colleagues with great affection and appreciation – affection for a wonderfully kind and generous man, with a great sense of humour, and appreciation for his dedication, insights and collegial manner. As noted by our colleague Maria Pilar Aguar Fernandez, “*Josema would spend endless hours trying to find a mathematical expression to life processes and interaction of chemicals with algae, plants and aquatic organisms. Josema has set an example as a researcher to the service of Europe and to the society; as a researcher he was committed to finding a solution to multiple unknowns emerging in our world and committed to give a better life and knowledge to the future generations*.”

Josema was so full of passion, enthusiasm and dedication for his scientific work that even when he was already in an advanced stage of his disease, he continued to work on running scientific projects, interacting daily with his colleagues at the Joint Research Centre and around the world.

Outside of work, Josema was devoted to his family, his wife Fernanda, and his two daughters, Giulia and Sara. In the words of his wife, Fernanda, *“Josema loved life, his family and his work and taught others to do the same. When you worked with him you had the feeling that everything would be successful and that the work would have been a great success.”* Indeed, thanks to Josema's inspiration and scientific legacy, the VCBA has moved from an original concept to a user-friendly software tool, taking us one step closer to our ultimate goal of an animal-free and mathematically-based framework for chemical safety assessment.

**Figure:**
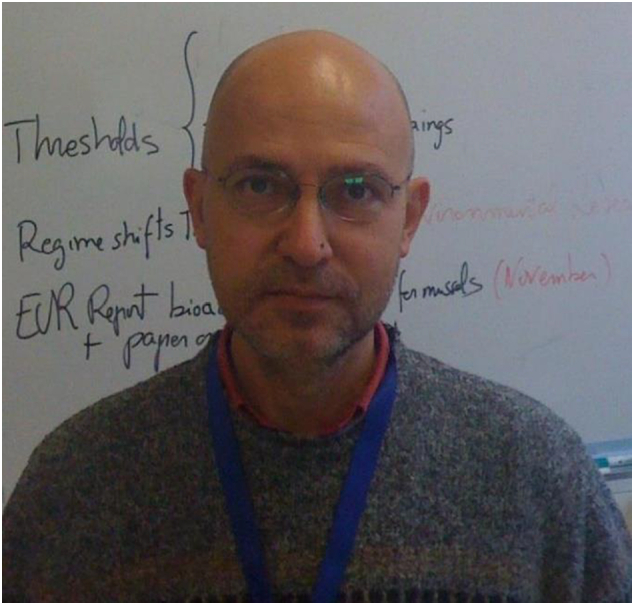

## Introduction to the Virtual Cell Based Assay and this Special Issue

The VCBA is a mathematical model designed to simulate the time-dependent fate and effects of chemicals in an *in vitro* test system (well-plate), including the free concentration in the test medium and the concentrations within cellular compartments. The outputs of the VCBA can be integrated into physiologically based kinetic (PBK) models in order to support the design of *in vitro* toxicity studies and support the chemical risk assessment process.

More specifically, the VCBA is an integrated model composed of: [1] a fate and transport model; [2] a cell partitioning model; [3] a cell growth and division model; [4] a cell toxicity and effects model. The mathematical formulation of the original VCBA is described in the first paper within this special issue (J.M. Zaldívar Comenges et al.), and various extensions (inclusion of new compartments and applicability to additional cell lines) are described in the following paper (Worth et al.). The VCBA can be used in combination with PBK models to link *in vitro* kinetics to *in vivo* exposure (Paini et al., a), and applied to both single dose (Worth et al.), and repeated dose (Paini et al., b) exposure scenarios. The results can be used in risk assessment to link a dose to an effect or vice versa, using solely *in vitro* and *in silico* tools. Since the development of automated software tools is an important step in harmonising and expediting the chemical safety assessment process, the VCBA has been implemented into a KNIME workflow (J.V. Sala Benito et al.). Finally, taking stock of progress and offering a future perspective, opportunities and challenges posed by this line of research are outlined (Graepel and Lamon et al.). While some of these challenges will require considerable further *in vitro* experimentation, it can be seen from the papers in this special issue that considerable progress has been made in the past five years within the COSMOS project, not only in developing the computational models and tools, but also in making them available and deployable in a user-friendly form.

